# Processing of DNA Double-Strand Breaks by the MRX Complex in a Chromatin Context

**DOI:** 10.3389/fmolb.2019.00043

**Published:** 2019-06-07

**Authors:** Erika Casari, Carlo Rinaldi, Antonio Marsella, Marco Gnugnoli, Chiara Vittoria Colombo, Diego Bonetti, Maria Pia Longhese

**Affiliations:** Dipartimento di Biotecnologie e Bioscienze, Università degli Studi di Milano-Bicocca, Milan, Italy

**Keywords:** Mre11, Rad50, Xrs2/NBS1, Sae2/CtIP, Tel1/ATM, MRX complex, double-strand break, resection

## Abstract

DNA double-strand breaks (DSBs) are highly cytotoxic lesions that must be repaired to ensure genomic stability and avoid cell death. The cellular response to DSBs is initiated by the evolutionarily conserved Mre11-Rad50-Xrs2/NBS1 (MRX/MRN) complex that has structural and catalytic functions. Furthermore, it is responsible for DSB signaling through the activation of the checkpoint kinase Tel1/ATM. Here, we review functions and regulation of the MRX/MRN complex in DSB processing in a chromatin context, as well as its interplay with Tel1/ATM.

## Introduction

Chromosomal DNA double-strand breaks (DSBs) are potentially lethal DNA lesions that can form accidentally during DNA replication and transcription, or upon exposure to genotoxic agents, such as ionizing radiation or chemicals. Failure to repair them can result in loss of genetic information or cell death, whereas inaccurate repair can lead to chromosome rearrangements (Jackson and Bartek, 2009; Liu et al., 2012). Even though DSBs pose a significant threat to genome stability, DSBs are programmed recombination intermediates during gametogenesis or antigen-receptor diversity in lymphocyte development (Lam and Keeney, 2014; Arya and Bassing, 2017). In all cases, DSBs need to be repaired to preserve genomic integrity.

Eukaryotic cells possess two main mechanisms for repairing DSBs: non-homologous end-joining (NHEJ) and homologous recombination (HR). Repair by NHEJ requires the Ku70–80 heterodimer (hereafter referred to as Ku) that recruits the DNA ligase IV complex (Lig4/Dnl4 in *Saccharomyces cerevisiae*), which directly religates the two broken ends (Chang et al., 2017). By contrast, HR is a more complex process that uses DNA information stored in a homologous double-stranded DNA (dsDNA) as template to reconstitute any missing genetic information at the break site (Mehta and Haber, 2014; Kowalczykowski, 2015).

The key process in determining which pathway is used to repair DSBs is the initial processing of the DSB ends. While NHEJ requires little or no DNA end processing, HR is initiated by nucleolytic degradation of the 5′ terminated strands at both DNA ends by a concerted action of nucleases in a process termed DNA end resection (Bonetti et al., 2018). The preferential degradation of the 5′-terminated strands results in formation of 3′-ended single-stranded DNA (ssDNA) ends that are first coated by the Replication Protein A (RPA) complex. RPA is subsequently replaced by Rad51 to form a nucleoprotein filament that is used to search for a homologous dsDNA sequence (Kowalczykowski, 2015). Repair can then proceed via synthesis-dependent strand annealing or the canonical recombination pathway that involves formation of a double Holliday junction (Mehta and Haber, 2014).

Extended resection of the DSB ends not only commits DSB repair to HR, but it makes the DNA ends non-ligatable by NHEJ. In vegetatively growing cells, HR uses the sister chromatid as repair template and this restricts recombination to the S and G2 phases of the cell cycle when the sister chromatid is available. This cell-cycle control of recombination is based on activation of key resection proteins by cyclin-dependent kinase (CDK)-catalyzed phosphorylation events (Aylon et al., 2004; Ira et al., 2004; Huertas et al., 2008; Chen et al., 2011).

The evolutionarily conserved Mre11-Rad50-Xrs2/NBS1 complex (MRX in *S. cerevisiae*, MRN in humans) recognizes, signals and initiates repair of DSBs. MRX is rapidly recruited to DSBs, where it has structural and enzymatic activities to initiate DSB resection and to maintain the DSB ends tethered to each other for their repair (Syed and Tainer, 2018). MRX also recruits and activates the checkpoint protein Tel1 (ATM in mammals) to coordinate DSB repair with cell cycle progression (Villa et al., 2016). Germline hypomorphic mutations of human MRN complex components are associated with Ataxia Telangiectasia-like disorder (ATLD), Nijmegen Breakage Syndrome (NBS) and NBS-like disorder, which are characterized by cellular radiosensitivity, immune deficiency and cancer predisposition (O'Driscoll, 2012). Here we review structure, functions and regulation of the MRX complex in sensing, signaling and processing DSBs within a chromatin context, focusing mainly on the work done in the budding yeast *S. cerevisiae*.

## Structural and Biochemical Properties of MRX

In both yeast and mammals, the MRX complex exists as a hetero-hexameric assembly, in which the Mre11 subunit interacts independently with both Rad50 and Xrs2 (NBS1 in mammals), and dimerizes with itself. Mre11 has five phosphodiesterase motifs in the N-terminal region and exhibits 3′-5′ dsDNA exonuclease and ssDNA endonuclease activities *in vitro* (Bressan et al., 1998; Paull and Gellert, 1998; Trujillo et al., 1998; Usui et al., 1998). The Sae2 protein (CtIP in mammals) stimulates Mre11 endonuclease activity to cleave the 5′-terminated DNA strands at both DSB ends (Cannavo and Cejka, 2014;Reginato et al., 2017; Wang et al., 2017).

**Figure 1:**
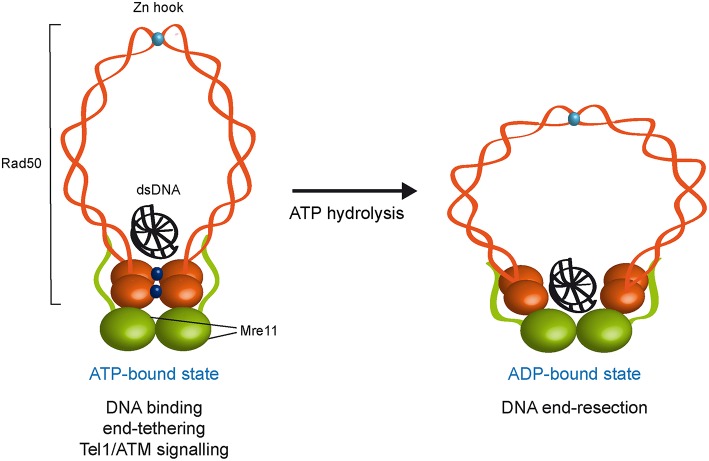
ATP- and ADP-bound state of the MRX complex. The Mre11 dimer (green) is bound to Rad50 dimer (orange) with a double-stranded DNA molecule located on the top surface of Rad50. The ATP-bound state of Rad50 supports DNA binding, end-tethering, and Tel1/ATM signaling, whereas it renders the dsDNA inaccessible to the Mre11 nuclease active sites and therefore negatively regulates Mre11 nuclease activity. ATP hydrolysis by Rad50 opens the complex to allow the Mre11 active sites to access DNA. Whether the ADP-bound state maintains an interlinked assembly is unknown. ATP molecules are indicated as blue dots. Zn^2+^ atoms are indicated as light blue dots. Xrs2 is not represented.

Rad50 is characterized by ATPase motifs at the N− and C− terminal regions of the protein, with the sequence in between forming two long coiled-coil domains that are separated by a zinc binding CXXC motif referred to as zinc hook (Syed and Tainer, 2018; Figure 1). The two ATPase motifs associate together to generate an ATP nucleotide binding domain and the coiled-coil domains fold back on themselves to form antiparallel intramolecular coiled coils (Hopfner et al., 2001; Moncalian et al., 2004; Williams et al., 2008; Figure 1). The zinc hook at the apex of the coiled-coil domains can form intralinked or interlinked complexes via tetrahedral coordination of a zinc^2+^ atom and the interlinked assembly can account for the MRX ability to maintain the DSB ends in close proximity (de Jager et al., 2001; Hopfner et al., 2002; Kaye et al., 2004; Lobachev et al., 2004; Wiltzius et al., 2005; Hohl et al., 2011; Nakai et al., 2011; He et al., 2012). Recently, crystal structure and X-ray scattering analyses of human RAD50 Zn-hook with a portion of the coiled-coil domain indicate the existence of a novel eukaryotic-specific interface that stabilizes Rad50 coiled coils in an intramolecular dimer assembly (Park et al., 2017), suggesting that the intralinked arrangement is the predominant form of the complex.

Several studies have shown that ATP binding and hydrolysis activities of Rad50 are crucial to regulate DNA binding, tethering and nuclease functions of the MRX complex. Structural studies of Mre11 in complex with Rad50 core domains from bacteria and archaea indicate that, upon ATP binding, Rad50 closes into a rigid conformation, in which the N- and C-terminal domains interact with each other and form a central groove that can accommodate dsDNA. This closed ATP-bound state of Rad50 renders dsDNA inaccessible to the Mre11 nuclease active site (Lammens et al., 2011; Lim et al., 2011; Williams et al., 2011; Möckel et al., 2012; Liu et al., 2016; Seifert et al., 2016). Point mutations that stabilize the ATP-bound conformation of Rad50 increase DNA binding, NHEJ and end-tethering (Deshpande et al., 2014), suggesting that MRX exerts these functions when it is present in the ATP-bound state. By contrast, in the ATP-free or hydrolyzed state, the Rad50 ATPase subunits are flexible and relatively open, suggesting that ATP hydrolysis drives the rotation of the two nucleotide binding domains of Rad50 and the disengagement of the Rad50 dimer that makes DNA accessible to the Mre11 nuclease active sites (Lammens et al., 2011; Lim et al., 2011; Williams et al., 2011; Möckel et al., 2012; Deshpande et al., 2014). Consistent with this hypothesis, biochemical analyses demonstrate that ATP hydrolysis by Rad50 is a prerequisite for Mre11/Rad50-mediated nuclease activity on dsDNA molecules (Paull and Gellert, 1999; Hopfner et al., 2000; Trujillo and Sung, 2001; Herdendorf et al., 2011). Altogether, these findings lead to a model whereby these ATP-driven transitions regulate the balance between MRX functions in NHEJ and end-tethering, which require ATP binding, and those in resection and HR, which require ATP hydrolysis (Figure 1).

Rad50 has a slow ATP hydrolysis rate (Herdendorf et al., 2011; Majka et al., 2012; Deshpande et al., 2017; Saathoff et al., 2018), suggesting that other proteins can promote its ATP hydrolysis activity within a cell. In *S. cerevisiae*, MRX is known to interact with Rif2, which is recruited to telomeric DNA ends and negatively regulates telomerase-mediated telomere elongation (Wotton and Shore, 1997; Levy and Blackburn, 2004; Hirano et al., 2009; Martina et al., 2012). Interestingly, Rif2, which is recruited to DSBs in a manner partially dependent on MRX, enhances ATP hydrolysis by Rad50 (Cassani et al., 2016). This observation, together with the finding that the lack of Rif2 increases the efficiency of both end-tethering and NHEJ (Cassani et al., 2016), suggests that Rif2 can regulate MRX ATP-driven transitions.

While Mre11 and Rad50 are conserved in bacteria and archaea, only eukaryotes possess Xrs2, which is the only MRX component that harbors a nuclear localization signal and is necessary for translocation of the Mre11-Rad50 subcomplex into the nucleus (Desai-Mehta et al., 2001; Tsukamoto et al., 2005). Localization of Mre11 into the nucleus in the absence of Xrs2 restores Mre11-Rad50 functions in DSB resection, hairpin resolution and meiotic recombination, but not in NHEJ and Tel1 activation (Oh et al., 2016), indicating an essential role for Xrs2 in these two latter processes. This finding is consistent with the observation that stimulation of the Mre11 endonucleolytic clipping activity by Sae2 requires Rad50 but not Xrs2 (Cannavo and Cejka, 2014).

By contrast, human NBS1 is required to promote MRE11 endonuclease activity on blocked DNA ends and hairpin substrates (Paull and Gellert, 1999; Deshpande et al., 2016). Using a reconstituted system, it has been recently shown that human NBS1 stimulates the MRE11-RAD50 nuclease by directly interacting with the MRE11 subunit and this stimulation requires CtIP phosphorylation (Anand et al., 2019). By contrast, in the absence of NBS1, MRE11-RAD50 subcomplex exhibits a weak nuclease activity that requires CtIP but not its phosphorylation (Anand et al., 2019). These findings lead to a model in which CtIP promotes MRE11 nuclease activity in a phosphorylation-dependent mode in the presence of NBS1 and in a phosphorylation-independent mode in the absence of NBS1, suggesting a role for NBS1 in restricting the MRE11-RAD50 nuclease to S and G2 phases of the cell cycle when CtIP is phosphorylated by CDKs.

## Role of MRX in DSB Resection

**Figure 2:**
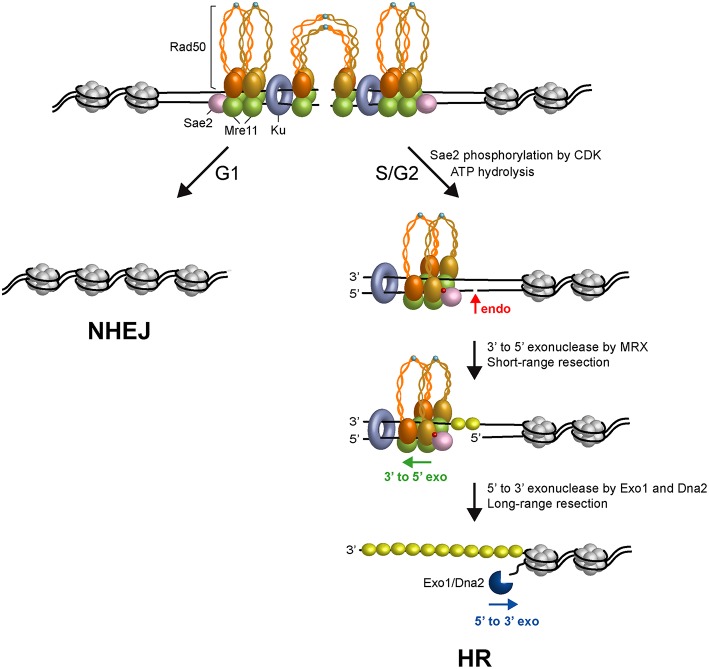
Model for initiation of DSB repair. Two ATP-bound MRX complexes are loaded onto both sides of the DSB, together with Ku and Sae2 proteins. The Rad50 subunits interact through the Zn-hook to form intralinked complexes. Owing to their proximity, the Zn-hook and coiled-coil domain may switch to form interlinked complexes that maintain the DSB ends tethered to each other. In the interlinked assembly, the Mre11 and Rad50 molecules are pictured separated from each other to visualize the DNA interruption. In G1, the DSB is repaired mainly by NHEJ because Sae2 is not phosphorylated, Rad50 is an ATP-bound state that blocks the Mre11 nuclease and Ku inhibits Exo1. In the S and G2 phases of the cell cycle, upon Sae2 phosphorylation by CDK and ATP hydrolysis by Rad50, Rad50 dimerization interface opens and dsDNA becomes accessible to the Mre11 nuclease active sites. Phosphorylated Sae2 then stimulates the Mre11 endonuclease to incise the 5′-terminated strands (red arrows) at Ku-bound DNA ends or adjacent to nucleosomes. MRX proceeds back toward the DSB end using the Mre11 3′-5′ exonuclease activity. Exo1 or Sgs1-Dna2 nuclease then can degrade DNA in the 5′-3′ direction. ssDNA generated by resection is coated by RPA to initiate HR. Phosphorylation is indicated as red dots. Zn^2+^ atoms are indicated as light blue dots. The Rad50 subunits belonging to a dimeric assembly are indicated with the same color (orange or gold). Xrs2 is not represented.

The obligate step that initiates all recombination pathways is the degradation of the 5′-terminated DNA strands at both DSB ends to generate 3′-ended ssDNA overhangs that catalyze homologous pairing and strand exchange (Bonetti et al., 2018). In both yeast and mammals, DNA end resection occurs in two main steps (Garcia et al., 2011; Shibata et al., 2014; Figure 2). In the first step, Sae2 activates the endonuclease activity of Mre11 within the context of the MRX complex to cleave the 5′-terminated DNA strands at both DSB DNA ends (Cannavo and Cejka, 2014). This step is followed by 3′-5′ nucleolytic degradation by Mre11 that proceeds back toward the DNA ends (Reginato et al., 2017; Wang et al., 2017). The MRX-Sae2 ensemble can degrade the 5′-terminated strands up to ~300 nucleotides away from the end and this processing is thus referred to as short-range resection. The resulting nick/gap provides an internal entry site for either Exo1 or the combined activities of the Sgs1 helicase and the Dna2 nuclease (Mimitou and Symington, 2008; Zhu et al., 2008; Cejka et al., 2010; Nicolette et al., 2010; Niu et al., 2010; Cannavo et al., 2013; Reginato et al., 2017; Wang et al., 2017). Exo1 and Dna2 are capable of resecting thousands of nucleotides in length in the 5′-3′ direction and this nucleolytic degradation is thus referred to as long-range resection.

### Short-Range Resection

Sae2 is known to be phosphorylated by multiple kinases, including CDKs and Mec1/Tel1 in a cell cycle- and DNA damage-dependent manner, respectively (Baroni et al., 2004; Cartagena-Lirola et al., 2006; Huertas et al., 2008; Manfrini et al., 2010). Using a reconstituted system, it has been shown that the ability of Sae2 to promote Mre11 endonuclease activity requires CDK-mediated Sae2 phosphorylation, and this control represents one of the key mechanisms that allow DSB resection to take place only during the S and G2 phases of the cell cycle when sister chromatids are available as repair templates (Huertas et al., 2008; Huertas and Jackson, 2009; Cannavo and Cejka, 2014; Anand et al., 2016). The phosphorylation state of Sae2 was shown to affect its oligomeric state that is critical for its activity (Kim et al., 2008; Fu et al., 2014; Andres et al., 2015; Davies et al., 2015). In particular, during the G1 phase of the cell cycle, Sae2 exists as unphosphorylated inactive soluble multimeric complexes (Cannavo et al., 2018). During S and G2 cell cycle phases or after DNA damage, phosphorylation at multiple Sae2 sites promotes formation of active Sae2 tetramers, which promote the Mre11 nuclease within the MRX complex (Cannavo et al., 2018). Furthermore, phosphorylation of the Sae2 C-terminus is necessary for a direct physical interaction between Sae2 and Rad50 (Cannavo et al., 2018). Since stimulation of Mre11 nuclease activity by Sae2 is dependent on ATP hydrolysis by Rad50 (Cannavo and Cejka, 2014; Wang et al., 2017), phosphorylated Sae2 might control the Mre11 nuclease by coupling ATP hydrolysis by Rad50 with Mre11 processing activity.

Genetic experiments have shown that MRX-Sae2-catalyzed cleavage is dispensable for resection of endonuclease-induced “clean” DSBs (Llorente and Symington, 2004), as Exo1 and Sgs1-Dna2 can directly access and resect the 5′-terminated strands of these DNA ends, although less efficiently. By contrast, MRX-Sae2-mediated cleavage is essential for removing hairpin-capped DSBs or protein blocks that render DNA ends refractory to Exo1- and Sgs1-Dna2-mediated resection (Lobachev et al., 2002; Neale et al., 2005). These end-binding factors can include trapped topoisomerases (Hoa et al., 2016) or Spo11, a meiosis-specific type II topoisomerase-like that generates programmed DSBs in meiosis by forming a covalent linkage between a conserved tyrosine residue and the 5′ end of the cleaved strand (Bergerat et al., 1997; Keeney et al., 1997). Spo11 is then removed endonucleolytically by Mre11, which introduces internal incisions at short distance from Spo11-bound DNA ends and releases short Spo11-attached oligonucleotides (Neale et al., 2005; Garcia et al., 2011).

Interestingly, using a reconstituted system, it has been shown that phosphorylated Sae2, or CtIP in humans, promotes the Mre11 nuclease within the MRX/MRN complex to cleave endonucleolytically the 5′-terminated DNA strand ~15–20 nucleotides away from a streptavidin block located at the end of a linear duplex DNA molecule (Cannavo and Cejka, 2014; Anand et al., 2016; Deshpande et al., 2016). Phosphorylated Sae2 was shown also to stimulate the MRX endonuclease activity on linear dsDNA substrates harboring either a streptavidin block or a catalytic inactive EcoRI restriction enzyme located at sites internal to the DSB end (Reginato et al., 2017; Wang et al., 2017). These findings suggest that any stable protein obstacle bound either internally or at the end of a DNA molecule can activate the 5′ DNA strand cleavage activity of MRX-Sae2.

The above observations raised the question of whether physiological protein blocks would also stimulate MRX-Sae2-catalyzed endonucleolytic cleavage. The Ku complex is rapidly recruited to DNA ends and protects them from degradation, particularly in the G1 phase of the cell cycle (Lisby et al., 2004; Clerici et al., 2008; Zierhut and Diffley, 2008). The lack of Ku partially restores DNA damage resistance in *sae2*Δ and *mre11* nuclease-deficient alleles (Clerici et al., 2008; Bonetti et al., 2010; Mimitou and Symington, 2010; Shim et al., 2010; Foster et al., 2011; Langerak et al., 2011), indicating that Ku bound to the DSB ends acts as a block to resection. Remarkably, Ku is as effective as a streptavidin block in stimulating the endonucleolytic cleavage by MRX in a manner that depends on phosphorylated Sae2 and ATP hydrolysis by Rad50 (Reginato et al., 2017; Wang et al., 2017). Furthermore, Ku shields DNA ends from the Mre11-catalyzed 3′-5′ degradation (Reginato et al., 2017; Wang et al., 2017). As MRX and Ku also promote NHEJ, these results support a model in which the presence of both MRX and Ku at the DSB ends in the G1 phase of the cell cycle first channels DSB repair into NHEJ (Figure 2). In S and G2 phases of the cell cycle, when Sae2 is phosphorylated by CDK and ATP hydrolysis by Rad50 is allowed, the presence of Ku at the DSB ends renders the 5′ DNA strand susceptible to endonucleolytic cleavage by MRX-Sae2 that directs the repair toward HR (Figure 2).

**Figure 3:**
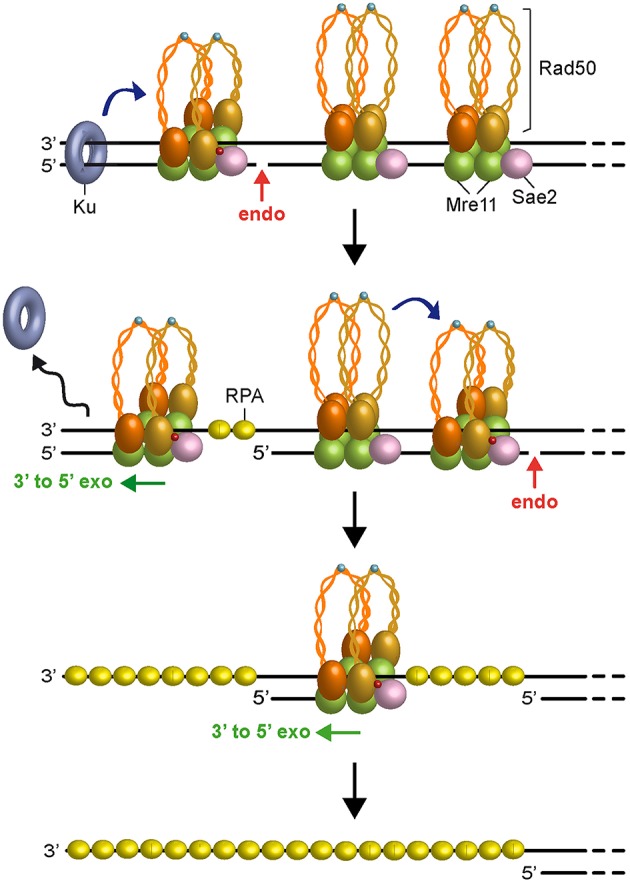
Model for short-range resection. Upon Sae2 phosphorylation and ATP hydrolysis by Rad50, MRX-Sae2 introduces an endonucleolytic cleavage (red arrow) on DNA ends that are bound by Ku, which acts as protein block to stimulate MRX-Sae2 cleavage (blue arrow). Mre11 3′-5′ exonuclease proceeds back toward the DSB end to generate ssDNA that removes Ku from DNA ends. Degradation proceeds by stepwise endonucleolytic incisions, in which one MRX complex can promote (blue arrow) cleavage by another MRX complex that is bound at an adjacent site. The endonucleolytic cleavage is followed by Mre11 3′-5′ exonucleolytic degradation of the DNA fragments between the incision sites. Zn^2+^ atoms are indicated as light blue dots. Phosphorylation is indicated as red dots.

In any case, as Ku preferentially binds dsDNA ends over ssDNA (Griffith et al., 1992), the 3′-5′ MRX-Sae2 processing activity should cause the removal of Ku from DNA ends (Mimitou and Symington, 2010; Langerak et al., 2011; Chanut et al., 2016), raising the possibility that other proteins could stimulate 5′ strand scission by MRX-Sae2 to overcome any obstacles present not only at DNA ends but also at sites internal to the DSB. Interestingly, similar to Ku, binding of the RPA complex to either partially resected DNA ends or terminal hairpin structures also stimulates MRX-Sae2 cleavage of the 5′ strand (Wang et al., 2017), suggesting that RPA can allow MRX-Sae2 to generate an entry site in case the long-range resection machinery is disassembled from partially resected DNA ends. Furthermore, a recent reconstitution of the *S. cerevisiae* short-range resection machinery has shown that the Mre11-Rad50 subcomplex and phosphorylated Sae2 can cleave a 5′-terminated DNA strand by stepwise incision without the requirement for a separate protein block (Cannavo et al., 2019). Altogether, these data lead to a model (Figure 3), in which Ku bound to DNA ends acts as a protein block to stimulate MRX-Sae2 cleavage. 3′-5′ Mre11 exonuclease proceeds back toward the DSB end and removes Ku from the DSB. Then, MRX-mediated degradation can proceed by stepwise endonucleolytic incisions, in which one MRX-Sae2 ensemble can act by its own as protein block to stimulate DNA cleavage by another MRX-Sae2 ensemble that is bound at adjacent sites internal to the DSB. The endonucleolytic cuts are followed by 3′-5′ exonucleolytic degradation by Mre11 exonuclease of the short DNA fragments between the incision sites.

Rad50 prevents degradation of the 3′-terminated DNA strand by limiting Mre11 exonuclease activity in an ATP-binding-dependent manner, thus explaining why the 3′-5′ exonuclease activity of Mre11 does not resect 3′-terminated strands at DSB sites (Cannavo et al., 2019). By contrast, phosphorylated Sae2 can partially overcome this inhibition by stimulating Mre11 exonuclease when ATP hydrolysis is allowed (Cannavo et al., 2019). However, because phosphorylated Sae2 also promotes the endonuclease of MRX, the exonuclease and endonuclease activities of MRX-Sae2 likely compete with each other.

### Long-Range Resection

Long-range resection can be carried out by either of two partially overlapping pathways, dependent on the enzymatic activities of Dna2 and Exo1 nucleases (Mimitou and Symington, 2008; Zhu et al., 2008; Cejka et al., 2010; Nicolette et al., 2010; Niu et al., 2010; Cannavo et al., 2013; Reginato et al., 2017; Wang et al., 2017). Inactivation of a single pathway results in only a minor resection defect, whereas major resection defects are only observed when both pathways are inactivated simultaneously (Mimitou and Symington, 2008; Zhu et al., 2008). While Exo1 is a dsDNA-specific exonuclease capable to degrade 5′-terminated DNA strands within a duplex DNA molecule (Tran et al., 2002), Dna2 is loaded on ssDNA ends and degrades them endonucleolytically, resulting in products of ~5–10 nucleotides in length (Kao et al., 2004). Dna2 resection activity requires an helicase activity that is provided by Sgs1 in yeast and by either BLM or WRN in human cells (Zhu et al., 2008; Sturzenegger et al., 2014; Pinto et al., 2016). In both yeast and mammals, Dna2 was shown to stimulate degradation of long ssDNA molecules by acting as a ssDNA translocase with 5′-3′ polarity (Levikova et al., 2017; Miller et al., 2017). This finding suggests that Sgs1 unwinds DNA in a 3′-5′ direction to provide Dna2 with ssDNA, and Dna2 translocates in a 5′-3′ direction to degrade the unwound 5′-terminated ssDNA strand.

In addition to provide an entry site for Dna2 and Exo1, MRX has also a structural role in promoting their resection activity, thus explaining why the resection defect of *mre11*Δ cells is more severe than that of *sae2*Δ or *mre11* nuclease defective mutants. Biochemical reconstitution experiments in both yeast and mammals have shown that MRX enhances the ability of Sgs1 to unwind dsDNA independently of Mre11 nuclease, possibly by increasing Sgs1 association to DNA ends (Cejka et al., 2010; Nicolette et al., 2010; Niu et al., 2010; Nimonkar et al., 2011; Cannavo et al., 2013). Furthermore, MRX/MRN enhances both the affinity to DNA ends and the processivity of Exo1 (Cejka et al., 2010; Nicolette et al., 2010; Niu et al., 2010; Nimonkar et al., 2011; Cannavo et al., 2013). Although Exo1 is a processive nuclease *in vitro*, single-molecule fluorescence imaging has shown that RPA strips Exo1 from DNA (Myler et al., 2016), implying that efficient resection requires multiple cycles of Exo1 rebinding at the same DNA end. Interestingly, MRX was shown to possess a weak ATP-dependent unwinding activity on dsDNA (Paull and Gellert, 1999; Cannon et al., 2013), which was proposed to be dependent on a rotation of the Rad50 nucleotide-binding domains (Liu et al., 2016). The recent identification of the hypermorphic *mre11-R10T* mutation, which increases Exo1 resection activity, has allowed us to demonstrate that this strand-separation function of MRX is important to stimulate Exo1 resection activity (Gobbini et al., 2018). In fact, molecular dynamic simulations have shown that the capping domains of wild type Mre11 dimer rapidly interact with the DNA ends and cause a partial unwinding of the dsDNA molecule, whereas the mutant Mre11-R10T dimer undergoes an abnormal rotation that leads one of the capping domain to wedge in between the two DNA strands and to persistently melt the dsDNA ends (Gobbini et al., 2018).

## Role of MRX in Tel1/ATM Activation

In both yeast and mammals, MRX is necessary for activation of the protein kinase Tel1/ATM (Carson et al., 2003; Uziel et al., 2003; Lee and Paull, 2004), which is a member of a serine/threonine protein kinase family with an N-terminal HEAT repeat domain and C-terminal kinase domain (Ciccia and Elledge, 2010; Gobbini et al., 2013). Mutations in the ATM gene are associated with the human syndrome Ataxia Telangiectasia (AT), whose clinical phenotypes are similar to those of ATLD and include neurodegeneration, sensitivity to IR, immunodeficiency, premature aging, radiosensitivity and predisposition to cancer (Shiloh and Ziv, 2013; Rothblum-Oviatt et al., 2016).

The exact mechanism of Tel1/ATM activation by MRX/MRN is mechanistically poorly understood. Indeed, in both yeast and mammals, MRX is required to recruit Tel1/ATM to DSBs through direct interaction between the N-terminal HEAT domain of Tel1/ATM and the C-terminal domain of the Xrs2/NBS1 subunit (Nakada et al., 2003; Falck et al., 2005; Lee and Paull, 2005; You et al., 2005). In *S. cerevisiae*, MRX and Tel1 association to DSBs is counteracted by Rif2, whose lack increases the association of MRX to DSBs in a Tel1-dependent manner (Hirano et al., 2009; Cassani et al., 2016). Co-immunoprecipitation experiments have shown that the C terminus of Xrs2 interacts with Rif2. As Tel1 also binds this Xrs2 region, Rif2 can limit Tel1 association to DSBs by interfering with MRX-Tel1 interaction (Hirano et al., 2009). Once Tel1 is recruited to DSBs by MRX, it plays a structural role in stabilizing the association of MRX to the DSB ends in a manner independently of its kinase activity (Cassani et al., 2016). This Tel1-mediated regulation of MRX retention on DNA ends is important to allow proper MRX-DNA binding that is needed for end-tethering and DSB repair (Cassani et al., 2016).

In any case, *in vitro* activation of human ATM by MRN requires ATP binding but not ATP hydrolysis (Lee et al., 2013), raising the possibility that MRX activates Tel1/ATM when it is present in the ATP-bound state. This hypothesis is supported by the identification of the separation-of-function *S. cerevisiae rad50-A78T* mutant allele, which specifically abolishes Tel1 activation without impairing MRX functions in DSB repair (Cassani et al., 2019). Molecular dynamics simulations have revealed that the mutant Mre11-Rad50^A78T^ subcomplex bound to ATP undergoes conformational rearrangements similar to those observed when wild type Mre11-Rad50 subcomplex is bound to ADP (Cassani et al., 2019), suggesting that failure of Mre11-Rad50^A78T^ to activate Tel1 is due to the inability of the mutant complex to maintain the closed conformation.

In *S. cerevisiae*, the lack of Sae2 increases MRX and therefore Tel1 persistence at DSBs (Lisby et al., 2004; Clerici et al., 2006, 2014). *mre11-nd* cells also exhibit persistent MRX and Tel1 association at DSB ends (Lisby et al., 2004; Yu et al., 2018; Colombo et al., 2019). These findings suggest that MRX-Sae2 processing activity contributes to eliminate MRX bound to DNA ends and this MRX displacement limits Tel1 signaling activity. However, *sae2*Δ cells, but not *mre11-nd* cells, exhibit increased accumulation of the Rad9 protein at DSBs and enhanced activity of the Rad53 checkpoint kinase, both of which inhibit the resection activity of Dna2-Sgs1 and Exo1 (Usui et al., 2001; Bonetti et al., 2015; Ferrari et al., 2015; Yu et al., 2018; Colombo et al., 2019). Mutations that decrease either MRX/Rad9 association to DSBs or Rad53/Tel1 signaling restores DNA damage resistance in Sae2-deficient cells (Bonetti et al., 2015; Chen et al., 2015; Ferrari et al., 2015; Gobbini et al., 2015; Puddu et al., 2015; Yu et al., 2018). These findings indicate that Sae2 has an Mre11 nuclease-independent function in resection that counteracts the inhibition that Rad9 and Rad53 exert on Exo1 and Dna2-Sgs1. The identification of the *sae2-ms* allele, which upregulates MRX and Tel1 signaling activities at DSBs but does not cause increased Rad9 association at DSBs and persistent Rad53 activation, suggests that Sae2 functions in dampening MRX-Tel1 and Rad53 signaling activities can be uncoupled (Colombo et al., 2019). These findings lead to a model whereby Sae2 removes MRX and Tel1 from DNA ends by promoting Mre11 nuclease activity, whereas it limits Rad9 accumulation to DSBs independently of Mre11 nuclease activity. Both these Sae2 functions contribute to downregulate Rad53 activation, with the control of Rad9 association playing the major role in supporting DNA damage resistance and checkpoint activation (Colombo et al., 2019).

## DSB Resection in A CHROMATIN Context

DNA is packaged through histone and non-histone proteins into a higher order structure called chromatin, which raises the question as to how DNA end resection occurs in the context of chromatin. Chromatin surrounding DSBs undergoes extensive modification and several highly conserved nucleosome remodelers are recruited to DNA DSBs. While some of them deposit covalent modifications on histone tails to facilitate DNA damage signaling and recruitment of repair factor, others alter chromatin structure either by replacing canonical histones with histone variants or by moving or evicting nucleosomes (Hauer and Gasser, 2017). These latter functions are carried out by proteins that use the energy of ATP hydrolysis to translocate on dsDNA and to disrupt histone-DNA contacts by nucleosome sliding, eviction or histone exchange (Osley et al., 2007).

Chromatin immunoprecipitation experiments support nucleosome disassembly near DSBs in both yeast and human cells (Li and Tyler, 2016; Tsabar et al., 2016), suggesting that nucleosome eviction occurs during resection. A key question is whether nucleosomes are evicted prior to the onset of resection or whether chromatin remodelers help the resection machinery to navigate through chromatin, with nucleosome loss occurring as a consequence of nucleolytic degradation. Genome-wide studies in meiotic cells suggest that MRX-Sae2 catalyzes the endonucleolytic cleavage preferentially on an internucleosomal DNA region at +1 and +2 nucleosomes proximal to meiotic DSB ends (Mimitou et al., 2017). Furthermore, MRX-Sae2 endonucleolytically cleaves the 5′ DNA strand bordering a nucleosome (Wang et al., 2017), thus explaining the ~100-nucleotide incremental cleavages detected at endonuclease-induced DSBs in *sgs1*Δ *exo1*Δ cells (Zhu et al., 2008). Thus, if nucleosomes are evicted near a DSB, their removal might occur after Mre11-dependent incision of the 5′-terminated strands. Consistent with a coexistence of both nucleosomes and MRX bound at DSB ends, single-molecule imaging studies have shown that MRX can diffuse along dsDNA even in the presence of nucleosomes (Myler et al., 2017).

Interestingly, by using an *in vitro*-reconstituted chromatin assay, it has been shown that the presence of nucleosomes impedes resection by both Exo1 and Sgs1-Dna2, with Exo1-dependent resection much more strongly affected (Adkins et al., 2013). This finding suggests that nucleosome destabilization or removal occurs before nucleolytic processing by Exo1, with a constraint on resection length being how many nucleosomes are removed (Mimitou et al., 2017). In any case, removal of H2A-H2B dimers from nucleosomes was shown to enhance Exo1 activity (Adkins et al., 2013). Furthermore, biochemical and genetic evidence reveals that nucleosomes harboring H2AZ, an H2A variant that has been linked to DSB repair, are more accessible to Exo1 (Adkins et al., 2013). These findings suggest that ATP-dependent chromatin-remodeling enzymes promote Exo1-mediated resection *in vivo*.

Several chromatin remodelers are recruited to chromatin regions adjacent to DSBs and are candidates for nucleosome destabilization during DSB resection (Hauer and Gasser, 2017). Both the RSC and the SWI/SNF complexes appear to promote MRX association to DSBs and subsequent DSB processing by catalyzing eviction or mobilization of nucleosomes adjacent to a DSB (Chai et al., 2005; Shim et al., 2007; Wiest et al., 2017). Also the INO80 complex is recruited to DSBs and participates in eviction of nucleosomes to facilitate Rad51 nucleoprotein filament formation (Morrison et al., 2004; van Attikum et al., 2004, 2007; Tsukuda et al., 2009). Furthermore, two other remodelers have been shown to facilitate long-range resection. Both the SWR-C complex, which replaces the H2A/H2B dimers with H2A.Z in an ATP-dependent manner (Mizuguchi et al., 2004), and the Fun30/SMARCAD1 nucleosome remodeler promote Exo1-mediated degradation (Morillo-Huesca et al., 2010; Chen et al., 2012; Costelloe et al., 2012; Eapen et al., 2012; Adkins et al., 2013). Interestingly, the resection defect of *fun30*Δ cells is suppressed by elimination of Rad9, suggesting that Fun30 stimulates Exo1 resection activity by alleviating a Rad9-dependent chromatin barrier (Chen et al., 2012; Eapen et al., 2012). Finally, mammalian CHD1, which belongs to the chromodomain helicase DNA-binding CHD family of chromatin remodelers, is recruited to chromatin in response to DSBs in an MRE11-dependent manner and promotes the loading of CtIP onto damaged DNA (Kari et al., 2016).

## Conclusions

Work in the last years has advanced our understanding of the structure, biochemical activities, and regulation of the MRX complex. However, we still do not know at the mechanistic level how the functions of Sae2 and Rad50 ATPase integrate to regulate Mre11 nuclease activity, how the endonuclease activity of MRX is targeted locally, or how chromatin structure influence the MRX/Sae2-mediated DNA incision. Given the importance of this protein complex in ensuring genome stability and therefore in preventing carcinogenesis, answering these questions will be strongly relevant to human diseases.

## Author Contributions

MPL conceptualized the work. EC, CR, and MPL wrote the manuscript. AM, MG, CVC, and DB revised and edited the manuscript.

### Conflict of Interest Statement

The authors declare that the research was conducted in the absence of any commercial or financial relationships that could be construed as a potential conflict of interest.
